# The basic characteristics of extracellular vesicles and their potential application in bone sarcomas

**DOI:** 10.1186/s12951-021-01028-7

**Published:** 2021-09-17

**Authors:** Shenglong Li

**Affiliations:** 1 Department of Bone and Soft Tissue Tumor Surgery, Cancer Hospital of China Medical University, Liaoning Cancer Hospital & Institute, Shenyang, 110042 Liaoning Province China; 2 Department of Tissue Engineering, Center of 3D Printing & Organ Manufacturing, School of Intelligent Medicine, China Medical University (CMU), No. 77 Puhe Road, Shenyang North New Area, Shenyang, 110122 China

**Keywords:** Extracellular vesicles, Bone sarcomas, Cancer diagnosis, Cancer therapy, Invasion and metastasis

## Abstract

Bone sarcomas are rare cancers accompanied by metastatic disease, mainly including osteosarcoma, Ewing sarcoma and chondrosarcoma. Extracellular vesicles (EVs) are membrane vesicles released by cells in the extracellular matrix, which carry important signal molecules, can stably and widely present in various body fluids, such as plasma, saliva and scalp fluid, spinal cord, breast milk, and urine liquid. EVs can transport almost all types of biologically active molecules (DNA, mRNA, microRNA (miRNA), proteins, metabolites, and even pharmacological compounds). In this review, we summarized the basic biological characteristics of EVs and focused on their application in bone sarcomas. EVs can be use as biomarker vehicles for diagnosis and prognosis in bone sarcomas. The role of EVs in bone sarcoma has been analyzed point-by-point. In the microenvironment of bone sarcoma, bone sarcoma cells, mesenchymal stem cells, immune cells, fibroblasts, osteoclasts, osteoblasts, and endothelial cells coexist and interact with each other. EVs play an important role in the communication between cells. Based on multiple functions in bone sarcoma, this review provides new ideas for the discovery of new therapeutic targets and new diagnostic analysis.

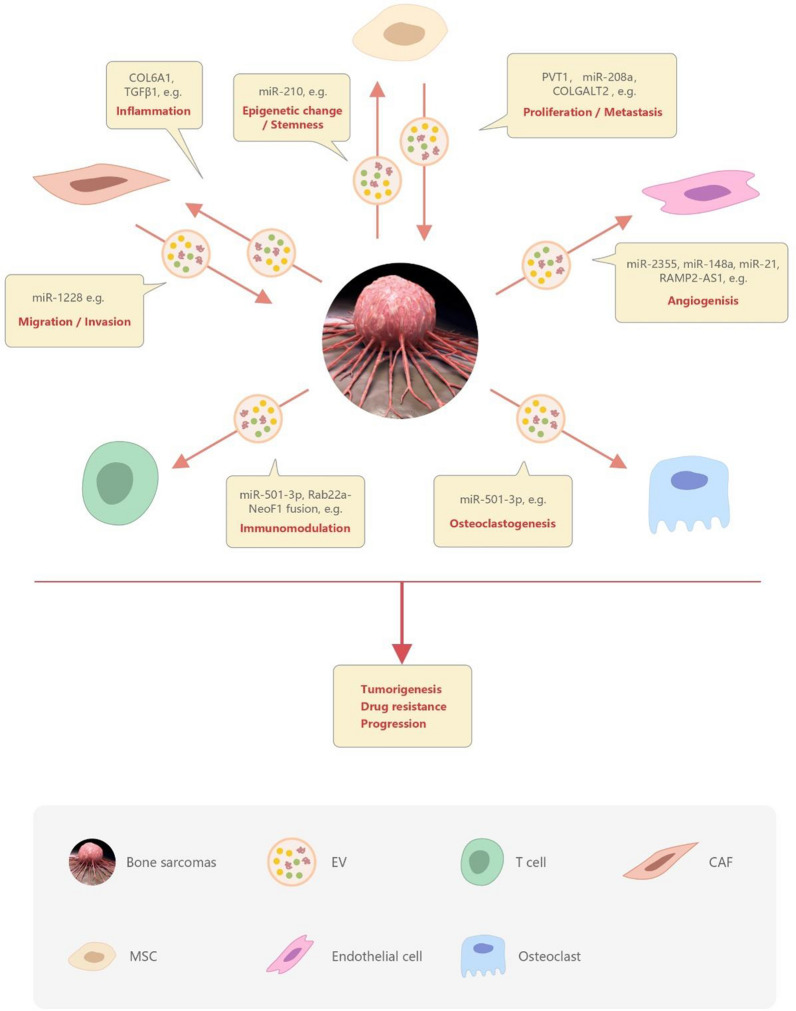

## Highlights

New role of EVs in cell–cell communication in bone sarcoma.

New clinical practicality and future application prospects of EVs.

EVs can be use as biomarker vehicles for diagnosis and prognosis in bone sarcomas.

Bone sarcomas cells derived EVs may influence angiogenesis, osteoclastogenesis, immunomodulation, drug resistance, invasion, and migration processes.

## Introduction

Bone sarcomas are malignant tumors that originates from mesenchymal tissue [1]. Osteosarcoma (OS) and chondrosarcoma are the most common malignant bone tumors, followed by Ewing sarcoma [1]. OS and Ewing sarcoma mainly occur in children and adolescents, while the incidence of chondrosarcoma increases with age [2, 3]. Osteosarcoma is the most common primary malignant bone tumor [4]. It mainly occurs in adolescents and children and reaches the second peak of incidence after old age [3]. Although neoadjuvant chemotherapy combined with surgical treatment can achieve a 5-year survival rate of 60–70%, for patients with relapsed and metastatic osteosarcoma, the original treatment method cannot produce effective therapeutic effects [5]. Chondrosarcoma is a malignant tumor derived from hyaline cartilage [6]. It can occur in any bone, but is common in the pelvis, humerus, femur, shoulder and ribs, and can occur at any age [6]. Patients with severe chondrosarcoma have a high mortality rate [7]. The treatment of chondrosarcoma includes aggressive surgical resection, systemic chemotherapy and targeted radiotherapy, but unfortunately, patients with chondrosarcoma often relapse and have a poor prognosis [7]. Ewing sarcoma mainly occurs in adolescents, and its malignant degree is high [8]. Recurrence and distant metastasis are the main causes of death [8]. Therefore, it is necessary to explore new treatment directions based on the development and metastasis mechanism of bone sarcomas.

Extracellular vesicles (EVs) are lipid bilayer nanovesicles secreted by cells, containing nucleic acids, proteins, lipids and other factors that maintain normal cell physiological functions and mediate cell-to-cell communication [9]. EVs are associated with multiple biological phenomena and are crucial for intracellular communication by transporting intracellular substances [10]. EVs are highly heterogeneous, and EVs secreted by different cells have different composition characteristics and functions. EVs have been regarded as wastes of cellular metabolism from the beginning to the current biological functions [11]. In addition to their important role in signal communication between cells, EVs are also widely involved in cell apoptosis, tumor development, angiogenesis, and immune response [12, 13]. Almost all cells secrete EVs under physiological and pathological conditions, and EVs can be found in blood, urine, saliva, and other body fluids [14]. EVs are widely observed in the tumor microenvironment [14]. They not only participate in the occurrence and development of tumors. Theoretically, tumor derived EVs are a dangerous "message in a bottle" for bone [15, 16]. EV plays a role in the regulation of bone remodeling activity and bone metastasis occurrence. They can modify the bone microenvironment, allowing the formation of osteolytic, osteosclerotic, and mixed metastasis [17]. However, the potential roles of EVs in the pathological exchange of bone cells between tumors and the bone microenvironment remain an emerging area [18]. The emerging evidence on EV functions in bone metastasis will facilitate the discovery of novel treatments [19].

In this review, we summarize the recent progress of the interaction between bone sarcoma and other cells in the tumor microenvironment through EVs, as well as the role of EVs as biomarker vehicles for diagnosis/prognosis and carriers for treatment in bone sarcomas. In particular, we discuss the role of these EVs in OS, Ewing sarcoma and chondrosarcoma, respectively. Compared with previous literature, we highlight the newly revealed role of EVs in cell–cell communication in bone sarcoma microenvironment, clinical practicality, and future application prospects.

## Biogenesis of EVs

EVs were first reported in 1946 as a platelet-derived procoagulant particles [20]. In 1983, a more detailed ultrastructural study found that during the differentiation of immature red blood cells, the fusion of multivesicular bodies (MVBs) with the cell membrane can also release similar vesicles and they are called exosomes [21].

**Fig. 1:**
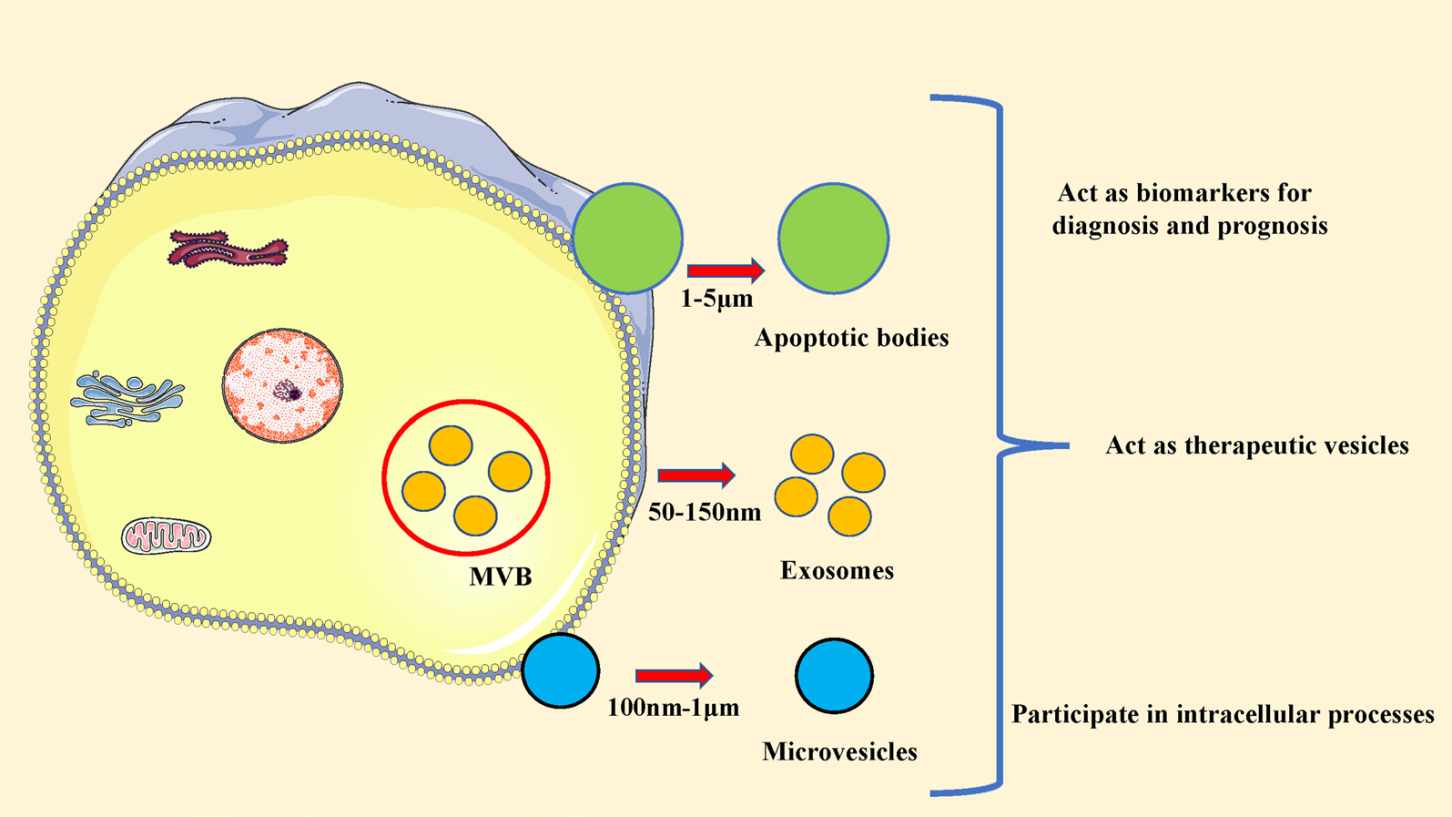
Types and biological roles of extracellular vesicle (EV). Multiple types of EVs are produced and range in size from nanometer through micrometer range. EVs can be roughly divided into three categories: microvesicles, exosomes and apoptotic bodies. EVs promote cell-to-cell communication and processes both locally and at a distance from their origination. EVs have the ability to serve as biomarkers. Additionally, their ability to target cells with specificity and incorporate therapeutics enables them to serve as therapeutic delivery vehicles. *MVB* multivesicular body

EVs can be roughly divided into three categories: Microvesicles produced by budding and division from the plasma membrane. The intraluminal vesicles released when MVBs fuse with the plasma membrane, namely exosome [22]. Apoptotic bodies released in the form of cell vesicles during cell apoptosis [23]. The size of EVs is usually used as the classification criteria: small vesicles < 150 nm are classified as exosomes, and those with 100 ~ 1000 nm or more are classified as micro-vesicles [24] (Fig. 1). There is still a lack of consensus on the nomenclature of extracellular vesicles. The International Society of Extracellular Vesicles (ISEV) encourages the use of "extracellular vesicles" as a general term and key word for all secreted vesicles [25].

The formation and secretion of EVs depends on the participation of endosomal sorting complex required for transport (ESCRT) [26]. As a consequence of their origin, exosomes from different cell types contain endosome-associated proteins (e.g., Rab GTPase, SNAREs, Annexins, and flotillin), some of which are involved in MVB biogenesis (e.g., Alix and Tsg101) [27]. The way in which virus-like microvesicles sprout on the surface or bind to the plasma membrane through MVBs to release ILVs plays an important role in the release of EVs [26]. At the same time, ESCRT ubiquitin-binding proteins HRS, STAM and Tsg101 are also involved in the sorting process of EVs [28]. Research on HRS protein has shown that the expression of dendritic cells defective in its expression, HGS gene (encoding HRS protein) knocked out HEK293 cell line and tumor cells, the release of exosomes is reduced, while silencing two genes that regulate HRS protein (HGS, STAM1) and the gene that regulates ESCRT-1 protein (TSG101) will reduce the release of EVs, which proves the important role of ESCRT protein in the biosynthesis and secretion of EVs [29].

EVs contain a variety of contents as follow.

Protein: Most of the proteins contained in EVs are shared by different types of EVs, such as tetrameric proteins (CD9, CD63, CD81 and CD82), 14–3-3 proteins, Major histocompatibility complex (MHC) molecules and specific stress proteins (heat shock proteins) and other cytoplasmic proteins; Endosome sorting complex 3 (ESCRT-3) binding protein required for transport. In general, EVs are very rich in cytoskeleton proteins, cytoplasmic proteins, heat shock proteins, cell membrane proteins, and proteins involved in vesicle transport, while there are fewer organelle proteins in the cell [30].

Lipids: EVs are different from their secreting cells in terms of lipid composition, and there may be a mechanism that can classify these specific lipid types into the vesicle [31].

Nucleic acid: EVs contain complete mRNA, mRNA fragments, long non-coding RNA (lncRNA), miRNA, ribosomal RNA (rRNA), different EVs may have different types and levels of these Nucleic acids [13]. EVs are loaded with content molecules of different types and contents to reflect the different states of parent cells. These substances also affect the properties and functions of EVs. For example, the content mRNA loaded by them is transferred horizontally through EVs and enters the recipient cells. It is translated into protein to change the biological state and function of the recipient cell [32], while miRNA can be stored in EVs in the blood circulation to avoid the degradation of RNAse, and then combine with immune cells to play an immunomodulatory effect. The system is an indispensable and important part in the occurrence and development of tumors [33].

## The detection methods of EVs

After the operation of separating and purifying extracellular vesicles, the morphology and purity of the extracellular vesicles need to be detected before sequencing or protein profiling of their contents (Table 1). This is a necessary condition to ensure the reliability of the later analysis data.

**Table 1:** The separation, enrichment and identification techniques for EVs

Classification	Detection technology	Aadvantages	Disadvantages
Separation and enrichment	Ultracentrifugation	Simple operation, can be used for large quantities of samples	The equipment required is expensive and time-consuming
	Density gradient centrifugation	The separation purity is improved, and the EV activity can be better maintained	Time-consuming, the osmotic pressure must be controlled when preparing inert gradient media
	Size exclusion chromatography	The separation purity is further improved, which saves time and can better maintain EV activity	The number of times the column is used and the amount of sample loaded are limited, and lipoproteins in some samples may be co-separated with EV
	Ultrafiltration	Simple operation, time-saving, can better maintain EV activity	Cannot filter out impurities smaller than the pore size of the filter membrane
			Affect purity and subsequent analysis
	Polyethylene glycol precipitation method	Reagents are easy to get, and the operation is simple	Time-consuming and susceptible to interference from other hydrophobic proteins, affecting the subsequent protein function analysis of EV
	Immunomagnetic bead sorting	Good specificity and high purity	Higher cost; limited by antibody preparation technology; epitope can be activated or blocked, affecting subsequent functional analysis
Identification	Electron microscope inspection	Can be used for EV morphological characterization	High equipment requirements, complicated sample preparation, dehydration, fixation, and dyeing may affect EV activity and high cost
	Nanoparticle tracking analysis technology	Real-time EV concentration and particle size distribution information can be obtained	Unable to distinguish the EV phenotype from the source, and the EV cannot be distinguished from particles of similar size; not suitable for heterogeneous samples, the light intensity signal of large particles can easily mask the signal of small particles
	western blot and Elisa	It can perform qualitative, semi-quantitative or quantitative analysis of the target protein with strong specificity	Only known proteins can be detected, limited by antibody preparation technology, the operation is cumbersome and time-consuming

### Electron microscope inspection

Electron microscopy is the gold standard for morphological detection of extracellular vesicles [34, 35]. The resolution of the transmission electron microscope is 1 ~ 3 nm, and the resolution of the scanning electron microscope is 5 nm. When observing an electron microscope sample, the diameter of extracellular vesicles can be measured. The immunogold labeling method can be used to label proteins on the surface of extracellular vesicles. Cryo-electron microscopy can observe the double-layer membrane structure of extracellular vesicles in order to distinguish between extracellular vesicles and other non-vesicular structures. Electron microscopy can reveal the structure of purified single extracellular vesicles or apoptotic bodies in tissues [36].

### Flow cytometry

Flow cytometry is an important method to analyze extracellular vesicles [34, 35]. Flow cytometric analysis sorts of particles according to the size of extracellular vesicles or the fluorescent signals carried. The size of extracellular vesicles is mainly analyzed by its scattered light, and the fluorescence carried by extracellular vesicles is mainly analyzed by the emission light of extracellular vesicles under laser excitation. The intensity of light scattered by extracellular vesicles is weak, sometimes lower than background noise. Extracellular vesicles can be labeled with fluorescent antibodies to be detected in flow cytometry. However, due to the small size of single extracellular vesicles, the abundance of labeled proteins on the surface is low, so the fluorescence intensity is lower than that of most streams. The resolution of the cytometer. Therefore, flow cytometry needs to distinguish the signal of extracellular vesicles under a lot of background noise.

### Nanoparticle tracking analysis technology

One of the most common methods for identifying EVs based on particle size is Nanoparticle Tracking Analysis Technology (NTA) [35]. This technology is to install a high-definition camera on an optical microscope, using the properties of light scattering and Brownian motion, through the Stokes-Instein equation (the movement speed of nanoparticles in their suspension per unit time and their own particle size There is a quantitative relationship between the viscosity of the solution and the temperature), the specific exosomes and microvesicles in the diameter range of 50 ~ 1000 nm are directly imaged and observed one by one, and the high-resolution particle size distribution data and concentration are obtained.

### Antibody-based identification method

Given that EV is produced in the cell membrane pathway, antibody targeting markers related to this pathway can be identified [35, 37]. These include the four transmembrane protein superfamily (CD9, CD63 and CD81), AIP1/Alix, TSG101 and CD326/EPCAM [38]. The method of identification can be western blotting.

### Fluorescence and Confocal Microscopy

EV can be labeled with lipophilic membrane-bound dyes (such as PKH67, DiD, etc.), or the sulfhydryl group on its surface can be used to label EVs [39, 40]. This technology cannot really visualize each EV, but it can be used to study whether the labeled EV can be taken up by cells.

## The role of EVs in bone sarcoma

**Fig. 2:**
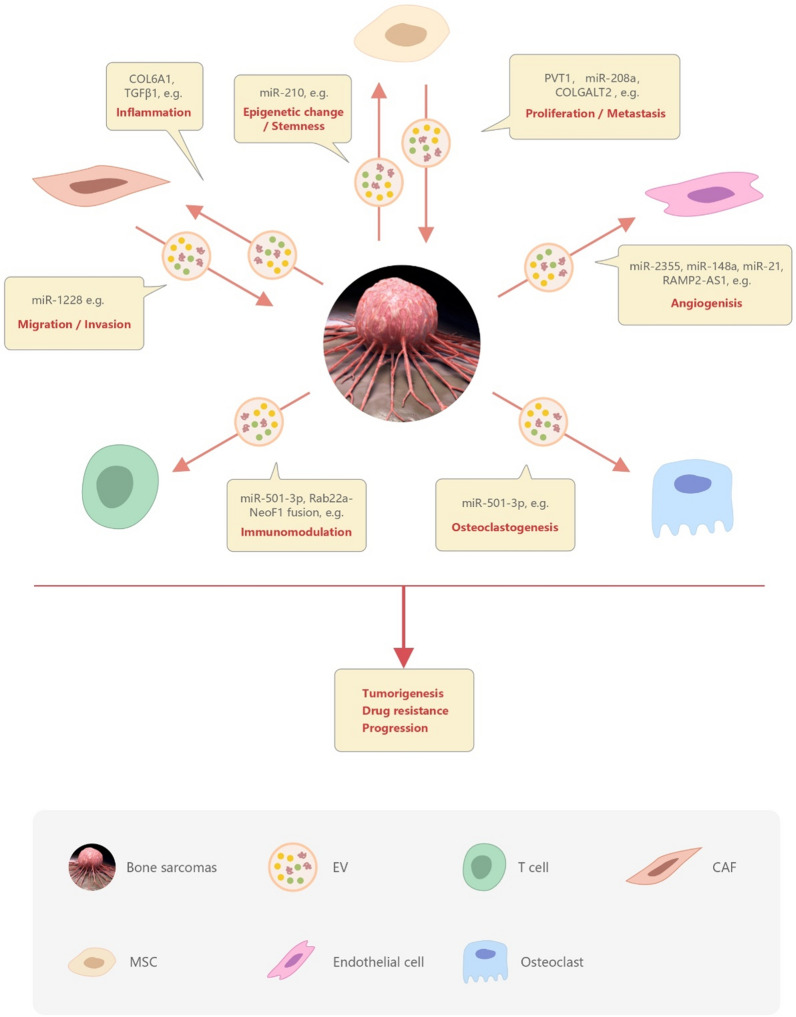
Role of extracellular vesicles in the communication between bone sarcomas cells and the tumor microenvironment. Bone sarcomas can interact with the surrounding cells through secretion and up-take of EVs. EVs participate in multiple pathways involved in tumor growth, progression, and metastatic process. EV-mediated crosstalk occurs through the trafficking of vesicle-associated components to endothelial cells, osteoclasts, T cells, CAFs, MSCs, and bone sarcomas cells. The loaded ingredients include some key miRs (e.g., miR-1228, miR-208a, and miR-501) and proteins (e.g., TGF-β, COL6A1, and COLGAL). Bone sarcomas derived EVs influence angiogenesis, osteoclastogenesis, immunomodulation, drug resistance, invasion, and migration processes. *CTCs* cancer stem cells, *CAFs* cancer-associated fibroblasts, *MSCs* mesenchymal stem cells, *EVs* extracellular vesicles

As a medium, EVs play a vital role in the communication between tumor cells and other cells in the tumor microenvironment (Fig. 2). Bone sarcomas cells can interact with surrounding cells through input/output of EVs. EV-mediated crosstalk occurs through the trafficking of vesicle-associated components to endothelial cells, osteoclasts, T cells, CTCs, CAFs, MSCs, and bone sarcomas cells. CAFs may transfer the ability of migration/invasion through EVs to bone sarcomas cells. Neighboring stem cells may transfer factors contributive to growth and metastasis. Bone sarcomas cells derived EVs may influence angiogenesis, osteoclastogenesis, immunomodulation, drug resistance, invasion, and migration processes. The roles of EVs in different types of bone sarcoma are as follow.

## The role of EVs in osteosarcoma

OS is the most common primary bone tumor, which occurs in 0.3 cases per 100,000 people [41]. Although diagnosis and treatment have improved in the past few decades, the survival rate for a considerable number of patients is still very low [3]. Therefore, one of the focuses of OS research is to better understand the metastatic process and how different factors regulate MTCT to promote metastatic spread. This will help determine treatment strategies for metastatic and refractory diseases, thereby improving survival.

Since EVs play a role in the tumorigenesis of many cancers and the role of these small extracellular vesicles in metastasis, it is not surprising that researchers in the OS field are studying the role of EVs in OS (Table 2).

**Table 2:** Summary of EVs studies in OS

Cargos	Parent cell	Target cell	Biological function	Reference
miR-206	BMSCs	OS cells	Cell proliferation, migration, invasion and apoptosis	[42]
Lnc-PVT1	BMSCs	OS cells	Tumor growth	[43]
miR-208a/PDCD4	BMSCs	OS cells	Promote OS progression	[44]
COLGALT2	AD-MSCs	OS cells	Promote OS progression	[45]
/	BMSCs	OS cells	Promote OS growth and metastasis	[46]
miR-135a-5p/LCP1	BMSCs	OS cells	Promote OS proliferation and metastasis	[47]
lnc-LIFR-AS1/miR-29a/NFIA	macrophage	OS cells	Cell proliferation, migration, invasion and apoptosis	[48]
miR-1228/SCAI	CAFs	OS cells		
			Cell migration and invasion	[49]
miR-151-3p/ CHL1/integrin 1β	CAFs	OS cells	Cell proliferation, migration, invasion	[50]
miR-101/BCL-6	AD-MSCs	OS cells		
			Tumor growth and metastasis	[51]
miR-675/CANN1	metastatic OS cells	Non-metastatic OS cells	Cell migration and invasion	[52]
Hic-5/Wnt /β-catenin	OS cells	OS cells	Cell proliferation and apoptosis	[53]
miR-1307/AGAP1	OS cells	OS cells	Cell proliferation, migration, and invasion	[54]
/	AXL up-regulated OS cells	AXL down-regulated OS Cells	Cell migration and invasion	[55]
immunomodulatory substances	OS cells	T cells	Reduce T cell activity	[56]
Tim-3	OS cells	Macrophage	Induce M2 polarization Tumor invasion and metastasis	[57]
miR-501-3p	OS cells	Osteoclast	Promote osteoclast production and aggravate bone loss	[58]
/	OS cells	CAFs	Cell differentiation	[61]
LINE-1	OS cells	MSCs	Epigenetic transformation	[63]
TGFB2	Metastatic OS cells	Macrophage	Regulate the cell signaling of tumor-associated macrophages	[64]
COL6A1	OS cells	CAFs	Activate CAF to promote OS transfer	[65]

Studies have shown that EVs derived from other cells can affect the function of osteosarcoma cells. Exosomes derived from bone marrow mesenchymal stem cells (BMSCs) could transport miRNA 206 (miR-206) to osteosarcoma cells. Mechanically, exosomal-miR-206 may inhibit the proliferation, migration and invasion of osteosarcoma cells by targeting TRA2B, and it may induce OS cell apoptosis [42]. Zhao et al. proved that BMSCs-derived exosomes encapsulated long non-coding PVT1 RNA and transported it to osteosarcoma cells, and the transported PVT1 promoted tumor growth by inhibiting ubiquitination and promoting ERG expression in osteosarcoma cells [43]. In addition, Qin et al. had shown that BMSC-derived exosomes miR-208a can improve the progression of osteosarcoma by targeting PDCD4 [44]. Wang et al. found that adipose-derived mesenchymal stem cells (AD-MSCs) exosomes can promote the progression of osteosarcoma by increasing the expression of COLGALT2 in osteosarcoma cells [45]. Human exosomes derived from BMSCs might promote the growth and metastasis of OS by promoting oncogenic autophagy [46]. Ge et al. demonstrated that BMSCs-derived exosomes LCP1 could promote bone proliferation and metastasis through JAK2/STAT3 pathway [47]. The targeting of miR-135a-5p/LCP1 axis might have potential in the treatment of OS [47]. The macrophage-derived exosomes lnc-LIFR-AS1 could promote the proliferation, invasion, and apoptosis of osteosarcoma cells through the miR-29a/NFIA regulatory axis [48]. Cancer-associated fibroblasts (CAFs) could spread exosomal miR-1228 by targeting SCAI, thereby promoting the invasion and migration of OS [49]. AD-MSCs could target BCL-6 to obtain miR-101-rich exosomes in OS cells, thereby inhibiting tumor growth and metastasis [50].

In addition, bone sarcomas can also secrete EVs to regulate their own tumor growth and metastasis. The results of Gong et al. study showed that metastatic OS cells could transfer exosomal miR-675 to non-metastatic cells and promoted cell migration and invasion by targeting CALN1 [51]. The inactivation of Hic-5 could inactivate Wnt/β-catenin signal through exosomal pathway, thereby inhibiting proliferation and inducing apoptosis of osteosarcoma cells [52]. The exosomal miR-1307 from OS cells promoted the proliferation, migration and invasion of OS cells by targeting AGAP1, and the miR-1307-AGAP1 axis might play an important role in the future treatment of OS [53]. In addition, the exosomal linc00852 associated with AXL up-regulated the proliferation, migration and invasion of OS cells, which was considered a novel tumor biomarker and a special therapeutic target for OS [54]. Towards endothelial cells, OS-derived exosomes promote osteoclasts differentiation and bone resorption activity, and these exosomes potentiated tube formation of endothelial cells and increased angiogenic markers expression. The molecular mechanisms underlying this process may include miR-148a and miR-21-5p [55, 56].

Studies have also shown that OS derived EVs might also participate in bone development by affecting the function of surrounding cells. Compared with normal osteoblasts, exosomes from osteosarcoma contain immunomodulatory substances, which could reduce the proliferation rate of T cells and promote the regulatory phenotype T [57]. Osteoblast exosomes could also reduce T cell activity, but to a lesser degree than canine osteosarcoma (OSA) exosomes and do not promote the T regulatory phenotype [57]. OS-derived exosomes might induce M2 polarization of macrophages and promote the invasion and metastasis of tumors by Tim-3 [58]. Bone sarcomas derived exosomal miR-501-3p promoted osteoclast production and aggravate bone loss through the PTEN/PI3K/Akt signaling pathway [59]. The Rab22a-NeoF1 fusion protein is secreted into exosomes through its KFERQ-like motif binding to HSP90. Macrophages and cancer cells negative for the fusion gene absorb the protein, while the exosomal fusion protein Rab22a-NeoF1 could promote its receptor-negative cancer cells metastasize in mouse lungs through the activation of RhoA activation by the binding partner PYK2 of their donor cells [60]. OS-derived EVs could recapitulate the infiltration of myeloid cells into the lungs of naive mice, but it is not enough to promote OS metastasis [61]. Mazumdar et al. also proved that EVs derived from OS cells could cause cancer-related fibroblast/fibroblast differentiation [62]. The OS cell line was able to produce EVs fused with recipient cells, and under the conditions of starvation, high-level activation of survival pathways, migration, adhesion, and 3D enhancement of ball formation, enhance its ability to grow in anchors, thereby enhancing proliferation and survivability [63]. OS-exosome-mediated LINE-1 methylation was insufficient in MSCs, while the opposite effect was observed in osteoblasts, indicating that MSCs are sensitive to epigenetic transformation but not to osteoblasts [64]. The exosomes of metastatic osteosarcoma cells could regulate the cell signaling of tumor-associated macrophages, thereby promoting the M2 phenotype by producing TGFB2 and creating an immunosuppressive microenvironment that promotes tumors [65]. Zhang et al. proved that COL6A1 can be packaged in OS derived exosomes and activate CAFs to promote OS transfer [66].

### The role of EVs in Ewing sarcoma

Ewing sarcoma (EWS) is a malignant tumor commonly seen in children and adolescents [67]. The only prognostic factor for patients with recurrence is the recovery time. Those who relapse 2 years after the initial diagnosis have a relatively good prognosis [68]. The 5-year survival rate of patients with local recurrence is 13%-30%, but the prognosis of patients with systemic or other tumor recurrence is better [68]. Recent studies have shown that EVs also play an important role in the development of Ewing sarcoma.

Feo et al. had shown that the elimination of CD99 in EWS tumor cells leads to the production and release of exosomes. These exosomes could transfer their anti-tumor effects to neighboring tumor cells. This indicated that these exosomes are in the reversal of malignant tumors rather than initiation in the soil. An atypical new role was played in the process transfer seeding [69]. Ventura et al. confirmed that the delivery of exosomes through CD99-silenced cells was sufficient to inhibit Notch-NF-kB signaling via miR-34a to induce neural differentiation of recipient EWS cells [70]. Miller et al. proved that EWS-derived exosomes might be used as biomarkers to minimize the diagnosis of residual diseases in peripheral blood, and prompt people to further study their potential biological effects in modifying the microenvironment related to EWS [71]. Hypoxic exosomes promoted stems in EWS cells by providing enriched miR-210 that could down-regulate the apoptotic pathway, leading to cell survival and increasing sphere formation [72].

### The role of EVs in chondrosarcoma

Chondrosarcoma is a malignant tumor that originates from cartilage or cartilage-forming connective tissue [3]. The incidence of malignant bone tumors ranks second, second only to osteosarcoma. The clinical manifestations of most lesions (especially secondary) are slow development, long duration, mild symptoms, and good prognosis; a few lesions (especially primary) progress fast, short duration, severe symptoms, and poor prognosis [3]. Cheng et al. found that chondrosarcoma cell-derived exosomes carry lncRNA RAMP2-AS1 and regulate the angiogenic ability of HUVECs via acting as a ceRNA of miR-2355-5p to regulate VEGFR2 expression [73].

## EVs as biomarker vehicles for diagnosis and prognosis in bone sarcomas

EVs are widely present in almost all body fluids, containing nucleic acids, proteins, lipids, metabolites, etc. Under different cell sources and different physiological or pathological conditions, the composition and content of EVs content will change significantly, and the level of specific content will change. The detection can reflect the physiological and pathological state of cells, and has the potential of liquid biopsy markers. At present, a variety of research strategies have been used in the screening of EVs markers, each with its advantages and limitations, and seeking the best clinical research strategy is still the key to screening for markers with high application value.

Detection of PD-L1 and exosomal N-cadherin in the serum of OS patients could predict the progression of lung metastasis in OS patients [74]. Cambier et al. verified that a consistent excess of DNA sequences of repetitive elements associated with EVs indicates their potential use as biomarkers for OS [75]. Li et al. found that SENP1 derived from plasma exosomes could be used as a new independent prognostic indicator in the clinical application of OS [76]. Zhang et al. introduced the latest progress of EWS and the opportunities and challenges brought by the development of circulating exosomes as a diagnosis and monitoring of children and young adults in the EWS family (ESFT) source of development of biomarkers for treatment response in adult patients [77]. Samuel and colleagues had also shown that circulating EVs could be used as a source of minimally invasive and potential prognostic diagnostic biomarkers in pediatric patients with tumors [78]. In addition, compared with the control, CASC15 upregulation was observed in OS plasma exosomes, and the same expression was observed in OS tissues and cell lines [79]. Besides, 30 gene fusions related to cancer patients have been identified as events in EVs RNA and are more common in metastatic EVs [80]. Analysis strategies for serum exosomal miRNAs and mRNAs have been developed for OS patients with different chemotherapeutic responses [81]. Compared with OS patients with good chemotherapy response, 12 miRNAs in OS patients with poor chemotherapy response were up-regulated, while 18 miRNAs were significantly down-regulated [81].

## The application of EVs in bone sarcoma treatment

The use of EVs to treat human diseases has become a core issue in clinical medicine because of their ability to deliver biologically active substances to target cells. Therefore, EVs are regarded as natural nanocarriers with high therapeutic potential [42, 82–86].

Pan et al. showed that exosomes from cisplatin-resistant cells (CDDP) reduced the sensitivity of MG63 and U2OS cells to CDDP, inhibited cell apoptosis, and increased the levels of multidrug resistance-related protein 1 and P-glycoprotein expression [87]. In addition, exosomal hsa_circ_103801 could enhance the promoter function of exosomes and promote the chemoresistance of MG63 and U2OS cells to CDDP [87]. Wei et al. found that the prepared doxorubicin-loaded exosomes could be used as an excellent chemotherapeutic drug for the treatment of osteosarcoma in vitro [88]. Considering the tumor localization function of BM-MSCs, doxorubicin-loaded exosomes might be a novel candidate for targeted therapy of OS in future studies. In addition, multidrug-resistant OS cells could expand their ability to resist the effects of adriamycin on sensitive cells by transferring exosomes carrying MDR-1 mRNA and its P-glycoprotein product [89].

## Remaining concerns and future perspectives

EVs are widely found in organisms, and their biological functions are increasingly recognized [90, 91]. As a natural communication medium between cells, EVs are expected to be used to treat a variety of clinical diseases based on this feature [11]. In addition, due to their high bioavailability and low immunogenicity, they can be the best candidates for drugs and therapeutic molecular carriers [12, 92]. For example, heterologous exosomes released by mesenchymal stem cells are considered a reliable and safe source of therapeutic exosomes [93, 94]. Clinically, the use of autologous methods is not ruled out, because in this case, the possibility of exosomes containing potentially dangerous molecules is very small. However, the exosomes of the patient's plasma are dangerous. Because the molecules delivered by the plasma exosomes are considered to be some metabolic wastes of diseased tissues, they are likely to deliver some drugs with high toxic potential. These studies support the use of exosomes secreted by primary monocytes in peripheral blood as drug carriers. Of course, it is not to say that they are completely safe, but they are safer than plasma exosomes, which requires clinical research to determine the true therapeutic potential of EVs. However, the most likely problem to be solved is the processing of EVs content. To this end, the best results can be achieved by establishing “EVs factories” similar to cell therapy cell factories. Another issue that needs to be addressed is the mechanism by which EVs play a therapeutic role. The mechanism of the interaction between EVs and target cells may be membrane-membrane fusion or the delivery of vesicle contents in target cells. Of course, they themselves may trigger effects. For example, EVs secreted by different types of cells may preferentially target certain cell types depending on the composition of the membrane, thereby having different effects on our body. Nevertheless, the mechanism of how EVs play a therapeutic role and affect target cells remains to be elucidated. Whether it is direct modification of EVs or selection of different cell sources for EVs, the safety issues still exist. Therefore, the research in the next few years may focus on the research on the impact of EVs on the body. There is no doubt that these goals can only be achieved after careful and in-depth research on the content and characteristics of EVs that have not been used in clinical practice.

In the microenvironment of bone sarcoma, bone sarcoma cells, mesenchymal stem cells, immune cells, fibroblasts, osteoclasts, osteoblasts, and endothelial cells coexist and interact with each other [15, 95, 96]. EVs play an important role in the communication between cells. On the one hand, osteosarcoma cells secrete EVs to reach the recipient cells to promote tumor support properties. On the other hand, EVs derived from tumor microenvironment cells can help tumor growth and migration. EVs with specific cell membrane components and specific wraps of the source cells have great potential as diagnostic and prognostic markers. EVs have the above-mentioned multiple functions in bone sarcoma, providing new ideas for the discovery of new therapeutic targets and new diagnostic analysis.

In the future, technological advances in the purification and characterization of EVs are expected to better help the detection of EVs and the study of their biological characteristics, and its clinical application prospects in bone sarcoma will be broader. At least, future research can focus on the following aspects. First, the identification of the expression profile of EV-specific inclusions is helpful for machine learning to identify the occurrence and types of OS. Second, the mechanism of transmission of drug resistance or metastatic properties by EVs can be further explored from multiple dimensions. Third, which type of EV is most easily ingested by bone sarcoma is a question to be addressed for the development of targeted drug carriers.

## Data Availability

The data that support the findings of this study are available from the corresponding author upon reasonable request.

## Abbreviations

EVs: Extracellular vesicles
miRNAs: MicroRNAs
OS: Osteosarcoma
EWS: Ewing sarcoma
MVBs: Multivesicular bodies
ISEV: International Society of Extracellular Vesicles
ESCRT: Endosomal sorting complex required for transport
MHC: Major histocompatibility complex
lncRNA: Long non-coding RNA
rRNA: Ribosomal RNA
PEG: Polyethylene glycol
NTA: Nanoparticle Tracking Analysis
BMSCs: Bone marrow mesenchymal stem cells
ERG: ETS transcription factor ERG
PDCD4: Programmed cell death 4
AD-MSCs: Adipose-derived mesenchymal stem cells
COLGALT2: Collagen beta(1-O)galactosyltransferase 2
LCP1: Lymphocyte cytosolic protein 1
NFIA: Nuclear factor I A
CAFs: Cancer-associated fibroblasts
CHL1: Cell adhesion molecule L1 like
CALN1: Calneuron 1
AGAP1: ArfGAP with GTPase domain, ankyrin repeat and PH domain 1
COL6A1: Collagen type VI alpha 1 chain

## References

[1] Brownstein JM, DeLaney TF (2020). Bone sarcomas and desmoids. Hematol Oncol Clin North Am.

[2] Miwa S, Yamamoto N, Hayashi K, Takeuchi A, Igarashi K, Tsuchiya H (2019). Therapeutic targets for bone and soft-tissue sarcomas. Int J Mol Sci.

[3] Aran V, Devalle S, Meohas W, Heringer M, Cunha Caruso A, Pinheiro Aguiar D, Leite Duarte ME, Moura Neto V (2021). Osteosarcoma, chondrosarcoma and Ewing sarcoma: clinical aspects, biomarker discovery and liquid biopsy. Crit Rev Oncol Hematol.

[4] Zhang XB, Zhang RH, Su X, Qi J, Hu YC, Shi JT, Zhang K, Wang KP, Zhou HY (2021). Exosomes in osteosarcoma research and preclinical practice. Am J Transl Res.

[5] Gazouli I, Kyriazoglou A, Kotsantis I, Anastasiou M, Pantazopoulos A, Prevezanou M, Chatzidakis I, Kavourakis G, Economopoulou P, Kontogeorgakos V (2021). Systematic review of recurrent osteosarcoma systemic therapy. Cancers (Basel).

[6] Monga V, Mani H, Hirbe A, Milhem M (2020). Non-conventional treatments for conventional chondrosarcoma. Cancers (Basel).

[7] MacDonald IJ, Lin CY, Kuo SJ, Su CM, Tang CH (2019). An update on current and future treatment options for chondrosarcoma. Expert Rev Anticancer Ther.

[8] Zollner SK, Amatruda JF, Bauer S, Collaud S, de Alava E, DuBois SG, Hardes J, Hartmann W, Kovar H, Metzler M (2021). Ewing sarcoma-diagnosis, treatment, clinical challenges and future perspectives. J Clin Med.

[9] Song H, Zhao J, Cheng J, Feng Z, Wang J, Momtazi-Borojeni AA, Liang Y (2021). Extracellular Vesicles in chondrogenesis and Cartilage regeneration. J Cell Mol Med.

[10] Li S, Wang X (2021). The potential roles of exosomal noncoding RNAs in osteosarcoma. J CELL PHYSIOL.

[11] Abhange K, Makler A, Wen Y, Ramnauth N, Mao W, Asghar W, Wan Y (2021). Small extracellular vesicles in cancer. Bioact Mater.

[12] Ruan J, Miao X, Schluter D, Lin L, Wang X (2021). Extracellular vesicles in neuroinflammation: pathogenesis, diagnosis, and therapy. Mol Ther.

[13] de Voogt WS, Tanenbaum ME, Vader P (2021). Illuminating RNA trafficking and functional delivery by extracellular vesicles. Adv Drug Deliv Rev.

[14] Liu Y, Xia Y, Smollar J, Mao W, Wan Y (2021). The roles of small extracellular vesicles in lung cancer: molecular pathology, mechanisms, diagnostics, and therapeutics. Biochim Biophys Acta Rev Cancer.

[15] Cappariello A, Rucci N (2019). Tumour-derived extracellular vesicles (evs): a dangerous, “message in a bottle” for bone. Int J Mol Sci.

[16] Tao SC, Guo SC (2019). Extracellular vesicles in bone: “dogrobbers” in the “eternal battle field”. Cell Commun Signal.

[17] Rossi M, Battafarano G, D'Agostini M, Del Fattore A (2018). The role of extracellular vesicles in bone metastasis. Int J Mol Sci.

[18] Li S, Wang W (2021). Extracellular vesicles in tumors: a potential mediator of bone metastasis. Front Cell Dev Biol.

[19] Tamura T, Yoshioka Y, Sakamoto S, Ichikawa T, Ochiya T (2020). Extracellular vesicles in bone metastasis: key players in the tumor microenvironment and promising therapeutic targets. Int J Mol Sci.

[20] Chargaff E, West R (1946). The biological significance of the thromboplastic protein of blood. J Biol Chem.

[21] Pan BT, Johnstone RM (1983). Fate of the transferrin receptor during maturation of sheep reticulocytes in vitro: selective externalization of the receptor. Cell.

[22] Beltraminelli T, Perez CR, De Palma M (2021). Disentangling the complexity of tumor-derived extracellular vesicles. Cell Rep.

[23] Gould SJ, Raposo G (2013). As we wait: coping with an imperfect nomenclature for extracellular vesicles. J Extracell Vesicles.

[24] Caserta S, Ghezzi P (2021). Release of redox enzymes and micro-RNAs in extracellular vesicles, during infection and inflammation. Free Radic Biol Med.

[25] Aikawa E, Gardiner C, Hutcheson JD, Ochiya T, Osteikoetxea X, Pegtel M, Piper M, Quesenberry P, Schiffelers RM, Szabó TG (2013). International Society for Extracellular Vesicles: Second Annual Meeting, 17–20 April 2013, Boston, MA (ISEV 2013). J Extracell Vesicles.

[26] Lazar S, Goldfinger LE (2021). Platelets and extracellular vesicles and their cross-talk with cancer. Blood.

[27] Raposo G, Stoorvogel W (2013). Extracellular vesicles: exosomes, microvesicles, and friends. J Cell Biol.

[28] Juan T, Furthauer M (2018). Biogenesis and function of ESCRT-dependent extracellular vesicles. Semin Cell Dev Biol.

[29] Colombo M, Raposo G, Thery C (2014). Biogenesis, secretion, and intercellular interactions of exosomes and other extracellular vesicles. Annu Rev Cell Dev Biol.

[30] Adamo G, Fierli D, Romancino DP, Picciotto S, Barone ME, Aranyos A, Bozic D, Morsbach S, Raccosta S, Stanly C (2021). Nanoalgosomes: Introducing extracellular vesicles produced by microalgae. J Extracell Vesicles.

[31] Nguyen VVT, Witwer KW, Verhaar MC, Strunk D, van Balkom BWM (2020). Functional assays to assess the therapeutic potential of extracellular vesicles. J Extracell Vesicles.

[32] Sung BH, Parent CA, Weaver AM (2021). Extracellular vesicles: critical players during cell migration. Dev Cell.

[33] Nagelkerke A, Ojansivu M, van der Koog L, Whittaker TE, Cunnane EM, Silva AM, Dekker N, Stevens MM (2021). Extracellular vesicles for tissue repair and regeneration: evidence, challenges and opportunities. Adv Drug Deliv Rev.

[34] Jiang Z, Liu G, Li J (2020). Recent progress on the isolation and detection methods of exosomes. Chem Asian J.

[35] Negahdaripour M, Owji H, Eskandari S, Zamani M, Vakili B, Nezafat N (2021). Small extracellular vesicles (sEVs): discovery, functions, applications, detection methods and various engineered forms. Expert Opin Biol Ther.

[36] Zhao X, Lei Y, Zheng J, Peng J, Li Y, Yu L, Chen Y (2019). Identification of markers for migrasome detection. Cell Discov.

[37] Wang S, Khan A, Huang R, Ye S, Di K, Xiong T, Li Z (2020). Recent advances in single extracellular vesicle detection methods. Biosens Bioelectron.

[38] Thery C, Ostrowski M, Segura E (2009). Membrane vesicles as conveyors of immune responses. Nat Rev Immunol.

[39] Lugini L, Cecchetti S, Huber V, Luciani F, Macchia G, Spadaro F, Paris L, Abalsamo L, Colone M, Molinari A (2012). Immune surveillance properties of human NK cell-derived exosomes. J Immunol.

[40] Tian T, Zhu YL, Zhou YY, Liang GF, Wang YY, Hu FH, Xiao ZD (2014). Exosome uptake through clathrin-mediated endocytosis and macropinocytosis and mediating miR-21 delivery. J Biol Chem.

[41] Llobat L, Gourbault O (2021). Role of MicroRNAs in human osteosarcoma: future perspectives. Biomedicines.

[42] Zhang H, Wang J, Ren T, Huang Y, Liang X, Yu Y, Wang W, Niu J, Guo W (2020). Bone marrow mesenchymal stem cell-derived exosomal miR-206 inhibits osteosarcoma progression by targeting TRA2B. Cancer Lett.

[43] Zhao W, Qin P, Zhang D, Cui X, Gao J, Yu Z, Chai Y, Wang J, Li J (2019). Long non-coding RNA PVT1 encapsulated in bone marrow mesenchymal stem cell-derived exosomes promotes osteosarcoma growth and metastasis by stabilizing ERG and sponging miR-183-5p. Aging (Albany NY).

[44] Qin F, Tang H, Zhang Y, Zhang Z, Huang P, Zhu J (2020). Bone marrow-derived mesenchymal stem cell-derived exosomal microRNA-208a promotes osteosarcoma cell proliferation, migration, and invasion. J Cell Physiol.

[45] Wang Y, Chu Y, Li K, Zhang G, Guo Z, Wu X, Qiu C, Li Y, Wan X, Sui J (2020). Exosomes secreted by adipose-derived mesenchymal stem cells foster metastasis and osteosarcoma proliferation by increasing COLGALT2 expression. Front Cell Dev Biol.

[46] Huang Y, Liu W, He B, Wang L, Zhang F, Shu H, Sun L (2020). Exosomes derived from bone marrow mesenchymal stem cells promote osteosarcoma development by activating oncogenic autophagy. J Bone Oncol.

[47] Ge X, Liu W, Zhao W, Feng S, Duan A, Ji C, Shen K, Liu W, Zhou J, Jiang D (2020). Exosomal transfer of LCP1 promotes osteosarcoma cell tumorigenesis and metastasis by activating the JAK2/STAT3 signaling pathway. Mol Ther Nucleic Acids.

[48] Zhang H, Yu Y, Wang J, Han Y, Ren T, Huang Y, Chen C, Huang Q, Wang W, Niu J (2021). Macrophages-derived exosomal lncRNA LIFR-AS1 promotes osteosarcoma cell progression via miR-29a/NFIA axis. Cancer Cell Int.

[49] Wang JW, Wu XF, Gu XJ, Jiang XH (2019). Exosomal miR-1228 from cancer-associated fibroblasts promotes cell migration and invasion of osteosarcoma by directly targeting SCAI. Oncol Res.

[50] Zhang K, Dong C, Chen M, Yang T, Wang X, Gao Y, Wang L, Wen Y, Chen G, Wang X (2020). Extracellular vesicle-mediated delivery of miR-101 inhibits lung metastasis in osteosarcoma. Theranostics.

[51] Gong L, Bao Q, Hu C, Wang J, Zhou Q, Wei L, Tong L, Zhang W, Shen Y (2018). Exosomal miR-675 from metastatic osteosarcoma promotes cell migration and invasion by targeting CALN1. Biochem Biophys Res Commun.

[52] Sha L, Ma D, Chen C (2020). Exosome-mediated Hic-5 regulates proliferation and apoptosis of osteosarcoma via Wnt/beta-catenin signal pathway. Aging (Albany NY).

[53] Han F, Pu P, Wang C, Ding X, Zhu Z, Xiang W, Wang W (2021). Osteosarcoma cell-derived exosomal miR-1307 promotes tumorgenesis via targeting AGAP1. Biomed Res Int.

[54] Li Q, Wang X, Jiang N, Xie X, Liu N, Liu J, Shen J, Peng T (2020). Exosome-transmitted linc00852 associated with receptor tyrosine kinase AXL dysregulates the proliferation and invasion of osteosarcoma. Cancer Med.

[55] Raimondi L, De Luca A, Gallo A, Costa V, Russelli G, Cuscino N, Manno M, Raccosta S, Carina V, Bellavia D (2020). Osteosarcoma cell-derived exosomes affect tumor microenvironment by specific packaging of microRNAs. Carcinogenesis.

[56] Wang S, Ma F, Feng Y, Liu T, He S (2020). Role of exosomal miR-21 in the tumor microenvironment and osteosarcoma tumorigenesis and progression (Review). Int J Oncol.

[57] Troyer RM, Ruby CE, Goodall CP, Yang L, Maier CS, Albarqi HA, Brady JV, Bathke K, Taratula O, Mourich D (2017). Exosomes from Osteosarcoma and normal osteoblast differ in proteomic cargo and immunomodulatory effects on T cells. Exp Cell Res.

[58] Cheng Z, Wang L, Wu C, Huang L, Ruan Y, Xue W (2020). Tumor-derived exosomes induced M2 macrophage polarization and promoted the metastasis of osteosarcoma cells through Tim-3. Arch Med Res.

[59] Lin L, Wang H, Guo W, He E, Huang K, Zhao Q (2021). Osteosarcoma-derived exosomal miR-501–3p promotes osteoclastogenesis and aggravates bone loss. Cell Signal.

[60] Zhong L, Liao D, Li J, Liu W, Wang J, Zeng C, Wang X, Cao Z, Zhang R, Li M (2021). Rab22a-NeoF1 fusion protein promotes osteosarcoma lung metastasis through its secretion into exosomes. Signal Transduct Target Ther.

[61] Mazumdar A, Urdinez J, Boro A, Arlt MJE, Egli FE, Niederost B, Jaeger PK, Moschini G, Muff R, Fuchs B (2020). Exploring the role of osteosarcoma-derived extracellular vesicles in pre-metastatic niche formation and metastasis in the 143-B Xenograft mouse osteosarcoma model. Cancers (Basel).

[62] Mazumdar A, Urdinez J, Boro A, Migliavacca J, Arlt MJE, Muff R, Fuchs B, Snedeker JG, Gvozdenovic A (2020). Osteosarcoma-derived extracellular vesicles induce lung fibroblast reprogramming. Int J Mol Sci.

[63] Urciuoli E, Giorda E, Scarsella M, Petrini S, Peruzzi B (2018). Osteosarcoma-derived extracellular vesicles induce a tumor-like phenotype in normal recipient cells. J Cell Physiol.

[64] Mannerstrom B, Kornilov R, Abu-Shahba AG, Chowdhury IM, Sinha S, Seppanen-Kaijansinkko R, Kaur S (2019). Epigenetic alterations in mesenchymal stem cells by osteosarcoma-derived extracellular vesicles. Epigenetics.

[65] Wolf-Dennen K, Gordon N, Kleinerman ES (2020). Exosomal communication by metastatic osteosarcoma cells modulates alveolar macrophages to an M2 tumor-promoting phenotype and inhibits tumoricidal functions. Oncoimmunology.

[66] Zhang Y, Liu Z, Yang X, Lu W, Chen Y, Lin Y, Wang J, Lin S, Yun JP (2021). H3K27 acetylation activated-COL6A1 promotes osteosarcoma lung metastasis by repressing STAT1 and activating pulmonary cancer-associated fibroblasts. Theranostics.

[67] Hesla AC, Papakonstantinou A, Tsagkozis P (2021). Current status of management and outcome for patients with ewing sarcoma. Cancers (Basel).

[68] Koustas E, Sarantis P, Karamouzis MV, Vielh P, Theocharis S (2021). The controversial role of autophagy in Ewing sarcoma pathogenesis-current treatment options. Biomolecules.

[69] De Feo A, Sciandra M, Ferracin M, Felicetti F, Astolfi A, Pignochino Y, Picci P, Care A, Scotlandi K (2019). Exosomes from CD99-deprived Ewing sarcoma cells reverse tumor malignancy by inhibiting cell migration and promoting neural differentiation. Cell Death Dis.

[70] Ventura S, Aryee DN, Felicetti F, De Feo A, Mancarella C, Manara MC, Picci P, Colombo MP, Kovar H, Care A (2016). CD99 regulates neural differentiation of Ewing sarcoma cells through miR-34a-Notch-mediated control of NF-kappaB signaling. Oncogene.

[71] Miller IV, Raposo G, Welsch U, Prazeres da Costa O, Thiel U, Lebar M, Maurer M, Bender HU, von Luettichau I, Richter GH (2013). First identification of Ewing's sarcoma-derived extracellular vesicles and exploration of their biological and potential diagnostic implications. Biol Cell.

[72] Kling MJ, Chaturvedi NK, Kesherwani V, Coulter DW, McGuire TR, Sharp JG, Joshi SS (2020). Exosomes secreted under hypoxia enhance stemness in Ewing's sarcoma through miR-210 delivery. Oncotarget.

[73] Cheng C, Zhang Z, Cheng F, Shao Z (2020). Exosomal lncRNA RAMP2-AS1 derived from chondrosarcoma cells promotes angiogenesis through miR-2355-5p/VEGFR2 axis. Onco Targets Ther.

[74] Wang J, Zhang H, Sun X, Wang X, Ren T, Huang Y, Zhang R, Zheng B, Guo W (2020). Exosomal PD-L1 and N-cadherin predict pulmonary metastasis progression for osteosarcoma patients. J Nanobiotechnology.

[75] Cambier L, Stachelek K, Triska M, Jubran R, Huang M, Li W, Zhang J, Li J, Cobrinik D (2021). Extracellular vesicle-associated repetitive element DNAs as candidate osteosarcoma biomarkers. Sci Rep.

[76] Wang L, Wu J, Song S, Chen H, Hu Y, Xu B, Liu J (2021). Plasma exosome-derived sentrin SUMO-specific protease 1: a prognostic biomarker in patients with osteosarcoma. Front Oncol.

[77] Zhang P, Samuel G, Crow J, Godwin AK, Zeng Y (2018). Molecular assessment of circulating exosomes toward liquid biopsy diagnosis of Ewing sarcoma family of tumors. Transl Res.

[78] Samuel G, Crow J, Klein JB, Merchant ML, Nissen E, Koestler DC, Laurence K, Liang X, Neville K, Staggs V (2020). Ewing sarcoma family of tumors-derived small extracellular vesicle proteomics identify potential clinical biomarkers. Oncotarget.

[79] Zhang H, Wang J, Ren T, Huang Y, Yu Y, Chen C, Huang Q, Guo W (2020). LncRNA CASC15 is upregulated in osteosarcoma plasma exosomes and CASC15 knockdown inhibits osteosarcoma progression by regulating miR-338-3p/RAB14 Axis. Onco Targets Ther.

[80] Bao Q, Gong L, Wang J, Wen J, Shen Y, Zhang W (2018). Extracellular vesicle RNA sequencing reveals dramatic transcriptomic alterations between metastatic and primary osteosarcoma in a liquid biopsy approach. Ann Surg Oncol.

[81] Xu JF, Wang YP, Zhang SJ, Chen Y, Gu HF, Dou XF, Xia B, Bi Q, Fan SW (2017). Exosomes containing differential expression of microRNA and mRNA in osteosarcoma that can predict response to chemotherapy. Oncotarget.

[82] Min L, Shen J, Tu C, Hornicek F, Duan Z (2016). The roles and implications of exosomes in sarcoma. Cancer Metastasis Rev.

[83] Théry C, Witwer KW, Aikawa E, Alcaraz MJ, Anderson JD, Andriantsitohaina R, Antoniou A, Arab T, Archer F, Atkin-Smith GK (2018). Minimal information for studies of extracellular vesicles 2018 (MISEV2018): a position statement of the International Society for Extracellular Vesicles and update of the MISEV2014 guidelines. J Extracell Vesicles.

[84] Masaoutis C, Korkolopoulou P, Theocharis S (2019). Exosomes in sarcomas: tiny messengers with broad implications in diagnosis, surveillance, prognosis and treatment. Cancer Lett.

[85] Brennan M, Layrolle P, Mooney DJ (2020). Biomaterials functionalized with MSC secreted extracellular vesicles and soluble factors for tissue regeneration. Adv Funct Mater.

[86] Casadei L, Pollock RE (2020). Extracellular vesicle cross-talk in the liposarcoma microenvironment. Cancer Lett.

[87] Pan Y, Lin Y, Mi C (2021). Cisplatin-resistant osteosarcoma cell-derived exosomes confer cisplatin resistance to recipient cells in an exosomal circ_103801-dependent manner. Cell Biol Int.

[88] Wei H, Chen J, Wang S, Fu F, Zhu X, Wu C, Liu Z, Zhong G, Lin J (2019). A nanodrug consisting of doxorubicin and exosome derived from mesenchymal stem cells for osteosarcoma treatment in vitro. Int J Nanomedicine.

[89] Torreggiani E, Roncuzzi L, Perut F, Zini N, Baldini N (2016). Multimodal transfer of MDR by exosomes in human osteosarcoma. Int J Oncol.

[90] Slomka A, Mocan T, Wang B, Nenu I, Urban SK, Gonzales-Carmona M, Schmidt-Wolf IGH, Lukacs-Kornek V, Strassburg CP, Sparchez Z (2020). EVs as potential new therapeutic tool/target in gastrointestinal cancer and HCC. Cancers (Basel).

[91] Villata S, Canta M, Cauda V (2020). EVs and bioengineering: from cellular products to engineered nanomachines. Int J Mol Sci.

[92] Avni D, Avni O (2021). Extracellular vesicles: schistosomal long-range precise weapon to manipulate the immune response. Front Cell Infect Microbiol.

[93] Liu H, Chen Y, Yin G, Xie Q (2021). Therapeutic prospects of MicroRNAs carried by mesenchymal stem cells-derived extracellular vesicles in autoimmune diseases. Life Sci.

[94] Ryan ST, Hosseini-Beheshti E, Afrose D, Ding X, Xia B, Grau GE, Little CB, McClements L, Li JJ (2021). Extracellular vesicles from mesenchymal stromal cells for the treatment of inflammation-related conditions. Int J Mol Sci.

[95] Perut F, Roncuzzi L, Baldini N (2019). The emerging roles of extracellular vesicles in osteosarcoma. Front Oncol.

[96] Lan M, Zhu XP, Cao ZY, Liu JM, Lin Q, Liu ZL (2018). Extracellular vesicles-mediated signaling in the osteosarcoma microenvironment: Roles and potential therapeutic targets. J Bone Oncol.

